# The Influence of Vitamin D Metabolism on Gene Expression, Matrix Production and Mineralization During Osteoprecursor Cell-Based Bone Development

**DOI:** 10.14740/jem212w

**Published:** 2014-04

**Authors:** Shelley S. Mason, Sean S. Kohles, Shelley R. Winn, Randy D. Zelick

**Affiliations:** a Department of Biology, Portland State University, Portland, Oregon, USA; b Presently with the Department of Cell and Molecular Biology, Oregon Health and Science University, Portland, Oregon, USA; c Department of Restorative Dentistry, Division of Biomaterials and Biomechanics, Oregon Health and Science University, Portland, Oregon, USA; d Department of Molecular and Medical Genetics, Oregon Health and Science University, Portland, Oregon, USA

**Keywords:** Cell and tissue engineering, Osteoinduction, Osteogenic factors, Stem cells, Mesenchymal stromal cells, Cholecalciferol, Calcitriol

## Abstract

**Background:**

Multipotential precursor cell lines derived from human bone marrow, capable of differentiating into cartilage or bone, may provide a useful tissue development model for studying the regulation and metabolism of putative growth and differentiation factors necessary for tissue regeneration. In mammals, the process of bone development depends on the proliferation and differentiation of osteoblast lineage cells, and the subsequent synthesis and mineralization of bone extracellular matrix (ECM). Vitamin D metabolites play a pivotal role in bone and mineral homeostasis, and are positive factors on bone development. Recently, it was demonstrated that a human-derived engineered osteoblast precursor cell line (OPC1), derived from human bone marrow, can metabolize the parental precursor vitamin D_3_ (vitaD_3_) to the active steroid 1α,25-dihydroxyvitamin D_3_ (1,25OH_2_D_3_), and elicit an osteogenic response that results in the decrease in proliferation and increase in ECM synthesis during early bone development. The aim in this study is to characterize gene expression, matrix production and mineralization within a bone development model.

**Methods:**

We investigated whether vitaD_3_ influences bone ECM mineralization in the same manner as 1,25OH_2_D_3_ in confluent cultures of OPC1s. In addition, we explored the influence of vitamin D metabolites, in combination with other commonly used osteogenic factors, ascorbic acid, β-glycerophosphate, dexamethasone (dex) and recombinant human bone morphogenetic protein-2 (rhBMP-2) on the osteoinduction of OPC1.

**Results:**

It was demonstrated that OPC1 expresses the mRNA for the enzymatic equipment necessary to convert vitaD_3_ to 1,25OH_2_D_3_, as well as the mRNA expression of the catabolic enzyme known to regulate the concentration of active 1,25OH_2_D_3_. It was also demonstrated that mRNA expression for the vitamin D receptor (VDR) was influenced by both vitaD_3_ and 1,25OH_2_D_3_. Differential results using vitamin D metabolites in combination with ascorbic acid, β-glycerophosphate, dex and/or rhBMP-2 were observed in alkaline phosphatase (ALP) activity and calcium deposition, and mRNA expression of procollagen type I (pro*Col*I), osteocalcin (OC) and osteopontin (OP).

**Conclusions:**

Overall it was demonstrated that vitamin D in combination with osteogenic factors influences the temporal bone development sequence in a positive manner.

## Introduction

Most of the load-bearing demand placed on the human body is transduced by skeletal tissue, and the skeleton’s capacity to articulate is essential for mobility and locomotion [1]. Consequently, cartilage and bone defects due to trauma, disease and developmental abnormalities may result in severe disabling pain and immobility for millions of people worldwide [2]. Multipotential precursor cell lines derived from human bone marrow, capable of differentiating into cartilage or bone, may provide a useful tissue development model for studying the regulation and metabolism of putative growth and differentiation factors necessary for tissue regeneration.

There are several factors that need to be taken into consideration for *in vitro* model systems, such as cell source and acquisition, phenotypic stability, reproducibility and the incorporation of appropriate signaling molecules. One of the challenges in using mesenchymal stromal cells (MSCs), or progenitor cells isolated from bone marrow aspirates, is isolation sufficiency and expansion. This is due to the relatively low frequency at which these cells exist in the marrow stroma (reported to be in the range of 1/10,000 cells to 1/100,000 cells) [1, 3, 4]. In addition, MSCs have a finite capacity for self-renewal, and the potential to proliferate and differentiate diminishes with age. MSC isolates also tend to be heterogeneous, containing progenitor cells with varying levels of potency. By contrast, the osteoblastic precursor cell line (OPC1) is a conditionally immortalized cell line derived from human bone, and has been demonstrated to maintain phenotypic stability and a consistent pattern of differentiation for more than 70 passages, providing an ideal model for the study of osteoblast differentiation and bone development as an alternative to MSCs [5]. We have used the OPC1 in a previous study and have shown it to provide a consistent and reproducible culture system for evaluating bone development [6]. We were able to demonstrate the extra-endocrine metabolism of vitamin D_3_ (vitaD_3_) to 1α,25-dihydroxyvitamin D_3_ (1,25OH_2_D_3_) by OPC1, which resulted in decreased proliferation and increased alkaline phosphatase (ALP) activity and calcium deposition associated with extracellular matrix (ECM) bone maturation and mineralization.

In the early 1900s, vitamin D was the fourth identified essential vitamin, thus named vitamin “D” by convention. It was not until 50 years later that it was realized that vitamin D is metabolized and acts as a steroid hormone in an endocrine manner, a paradigm that has dominated the classic thought of vitamin D function until the end of the 20th century [7]. Recently, it has been demonstrated that a number of organ systems are influenced by vitamin D in an autocrine manner, that is, 1,25OH_2_D_3_ is being synthesized within those cells, and/or within adjacent cells (paracrine action). While the vitamin D endocrine system plays a principal role in the maintenance of calcium and phosphate homeostasis, extra-endocrine metabolism has been postulated to have cellular functions that include the regulation of proliferation, differentiation and apoptosis in many target tissues [8–11], including bone and cartilage.

VitaD_3_ is biologically inactive in physiologic concentrations until it is sequentially hydroxylated and the polarity is increased. VitaD_3_ is activated by two-sequential hydroxylations, first by the cytochrome P450 oxidase (CYP) 25-hydroxylase, CYP27A1 (mitochondrial) and CYP2R1 (microsomal), resulting in the metabolite 25-hydroxyvitamin D_3_ (25OHD_3_), and then to the active metabolite, 1,25OH_2_D_3_, by 25-hydroxyvitaminD_3_-1α-hydroxylase (CYP27B1) [10, 12–14].

1,25OH_2_D_3_ is the most active form of vitamin D because of its high affinity for the intranuclear vitamin D receptor (VDR), mediating its biological influence upon binding. VDR belongs to the nuclear hormone receptor family and serves as a ligand-dependent transcription factor (TF) that controls the expression of vitamin D associated target genes which include those associated with calcium homeostasis, cellular proliferation and differentiation, cell division and cycle arrest, and apoptosis [15–19]. Upon 1,25OH2D3 binding, VDR forms a heterodimer with the retinoid X receptor, which allows it to bind to vitamin D receptor elements (VDREs) in the promoter regions of target genes [8, 14, 17, 20]. Coupled with other TFs, this complex induces transcription of vitamin D responsive genes including those associated with the CYP-related enzymes required for vitamin D metabolism [14, 17, 20–22].

The action of vitamin D is limited by the catabolic enzyme 24-hydroxylase (CYP24A1), which results in a compound that is more soluble and has a substantially lower affinity for VDR [12, 14, 23]. CYP24A1 has been shown to hydroxylate both 25OHD_3_ and 1,25OH_2_D_3_ to form 24,25OH_2_D_3_ and 1,24,25OH_3_D_3_, respectively [17]. The main function of CYP24A1 is to regulate the circulating concentration of 1,25OH_2_D_3_ by inactivating it. The catabolic pathway of CYP24A1, collectively known as the C-24 oxidation pathway, starts with the 24-hydroxylation of 25OH_2_D_3_ or 1,25OH_2_D_3_ followed by sequential steps of hydroxylation that lead to the progressive loss of biological activity until vitamin D is converted to the water-soluble biliary excretory form, calcitroic acid [10, 12, 24]. 1,25OH_2_D_3_ induces and mediates CYP24A1 via an autocatalytic loop through VDRE located in the promoter region of the CYP24A1 gene [21]. Generally, basal expression of CYP24A1 is extremely low but the gene is highly induced by 1,25OH_2_D_3_ [11, 20]. Thus, the synthesis and degradation of 1,25OH_2_D_3_ is highly regulated, and the concentration is controlled at the cellular level.

Vitamin D affects many aspects of bone cell biology, and has been implicated in the regulation of both osteoblastic and osteoclastic activity influencing both resorptive and synthetic phases of bone remodeling [11]. In addition, 1,25OH_2_D_3_ has been demonstrated to regulate osteoblast and chondrocyte gene transcription, proliferation, differentiation and ECM maturation [11, 25]. VDR is nearly ubiquitously expressed, as almost all cells respond to 1,25OH_2_D_3_ [26], and it is known to regulate the function of more than 200 genes [13, 17–19].

In this study, our objective was to quantify the differentiation of the OPC1 as an osteoprogenitor along the osteoblastic lineage including the events associated with bone development in the presence of vitamin D metabolites, vitaD_3_ and 1,25OH_2_D_3_. Additional osteogenic factors included ascorbic acid, β-glycerophosphate, dexamethasone and bone morphogenetic protein-2 (BMP-2). Because *in vitro* temporal bone development encompasses three stages (proliferation, bone ECM maturation and mineralization) and vitamin D has been shown to significantly hamper OPC1 proliferation [6], confluent cultures were used in order to investigate the influence on bone ECM maturation and mineralization.

## Materials and Methods

### Vitamin D and OPC1 cell cultures

VitaD_3_ (cholecalciferol) and 1,25OH_2_D_3_ (calcitriol) were purchased (Sigma-Aldrich, St. Louis, MO), and maintained in 10 mM and 10 μM stock solutions in ethanol, respectively. All other reagents and chemicals were purchased from the same vendor unless otherwise indicated. Original OPC1 and rhBMP-2 were prepared and maintained locally [5].

Cells were cultured at populations of 2.5 × 10^5^ in 75 cm^2^ tissue culture flasks in alpha modified essential medium (α-MEM) with 5% fetal bovine serum (FBS). Once confluent, OPCs were plated in 12-well plates after trypsin-ethylenediaminetetraacetic acid (EDTA) enzymatic removal and counted with 0.4% trypan blue (1:1) on a hemacytometer. Cultures were then prepared in duplicate at a seeding density of 3.0 × 10^4^ cells/well in the initial bone medium (designated as BM-) with 200 mM L-glutamine and antibiotics until 85–90% confluent prior to adding experimental medium. Nine variations of medium were utilized in quadruplicates. BM-with 95% ethanol (EtOH), in an amount that facilitated the EtOH as a vehicle for vitamin D metabolite delivery, provided a negative control (group 1). The additional groups included: BM- supplemented with 1 μM vitaD_3_ (group 2); BM- supplemented with 10 nM 1,25OH_2_D_3_ (group 3); BM-supplemented with osteogenic factors (BM+) comprised of 50 μg/mL ascorbic acid, 10 mM β-glycerophosphate, 10 nM dexamethasone (dex) and 50 μg/mL of ascorbic acid phosphate (Wako Chemical, Osaka, Japan), provided as a positive control (group 4); BM+ supplemented with 1 μM vitaD_3_ (group 5); and BM+ supplemented with 10 nM 1,25OH_2_D_3_ (group 6). Enhanced supplementation groups were also prepared: BM+ supplemented with rhBMP-2 (BMP+) (group 7); BMP+ with 1 μM vitaD_3_ (group 8); and BMP+ supplemented with 10 nM 1,25OH_2_D_3_ (group 9). Fresh medium was added every 1 to 2 days.

### Bone matrix maturation

ALP activity was monitored, as it is known to be involved with ECM maturation and mineralization through increased phosphate concentration [27]. ALP levels associated with the OPC1 response to both vitaD_3_ and 1,25OH_2_D_3_ in BM−, BM+ and BMP+ were determined in duplicate at the previously indicated concentrations. ALP was measured every 7 days. At the end of the incubation, the cell layers were washed with cold DPBS and 500 μL 0.1% triton-X in DPBS, then stored at −80 °C until the end of each experimental period. ALP activity in the cell lysates was measured using ρ-nitrophenyl phosphate liquid substrate system at 37 °C for 30 min. Protein content was measured using a micro-volume spectrophometer system (Epoch; Biotek, Winooski, VT).

### Bone matrix mineralization

Calcium deposition was determined through a histochemical analysis of mineralization utilizing Alizarin Red S (ARS). ARS staining identified calcium-rich deposits made by the cells in culture. This approach is considered a functional *in vitro* endpoint reflecting advanced cell differentiation [28]. Every 7 days the cells were fixed in 10% neutral buffered formalin for 15 min followed by fixation in 100% cold methanol. Each well was rinsed thoroughly with cold DPBS, and stained with 40 mM of ARS (pH = 4.1), at room temperature for 20 min with gentle shaking. The cell layers were rinsed and observed both grossly and microscopically. For semi-quantification, ARS was extracted from the cells after incubating for 1 h at room temperature in 10% volume to volume (v/v) acetic acid with gentle shaking, and then the residual dye was further extracted by scraping the cells and heating at 85 °C on a heating block with a layer of mineral oil to prevent evaporation. The samples were neutralized with 10% (v/v) ammonium hydroxide and the extracts were read at 405 nm on a microplate-reader (Cary 50; Varian Australia Pty. Ltd). The samples were compared to a serially diluted ARS standard. The resulting data were normalized with total protein content measured using a micro-volume spectrophometer system (Epoch; Biotek, Winooski, VT).

### Gene sequencing

Quantitative real-time, reverse transcription polymerase chain reaction (RT-PCR) analysis techniques were used to characterize the activity of the genes associated with vitamin D metabolism: CYP24A1, CYP27A1, CYP27B1 and VDR [16, 29]. In addition, RT-PCR was used to detect levels of osteopontin (OP), procollagen type I (pro*Col*I) and osteocalcin (OC). These noncollagenous and collagenous proteins are associated with hydroxyapatite binding and pre-collagen synthesis, respectively, during bone development [5]. The oligionucleotide RT-PCR primer sequences (Table 1) were purchased from a commercial source (Intergrated DNA Technologies, Coralville, IA). Briefly, messenger ribonucleic acid (mRNA) was isolated using 500 μL of a chemical reagent (TRIzol, Life Technologies, Grand Island, NY) per well of the 12-well plates. Samples were collected every 7 days and stored at −80 °C. The mRNA was purified according to the reagent manufacturer’s specifications and the concentration was determined in units of μg/mL by spectrophotometric absorbance of light at wavelengths of 260 nm and 280 nm (A_260_/A_280_) (Epoch, Biotek, Winooski, VT). The complementary deoxyribonucleic acid (cDNA) was synthesized from 2 μg of the mRNA using a high capacity reverse transcriptase kit (Applied Biosystems, Life Technologies) according to manufacturer’s instructions. Aliquots of the total cDNA were amplified in each PCR using fast green master mix (SYBR, Applied Biosystems, Life Technologies) with the acquired primers (Table 1). This amplification was performed in a quantitative thermocycler (Rotor-Gene Q, Qiagen, Germantown, MD) for 45 cycles (30 sec denaturation at 95 °C, 45 sec annealing at 58 °C and 60 sec extension at 72 °C).

### Microscopic imaging

Microscopic images were taken using an advanced transmitted light inverted microscope and accompanying software (EVOS-XL and EVOS3, Advanced Microscopy Group, Bothell, WA). Images were processed using an open source, image-processing package (FIJI, ImageJ v1.47d, National Institutes of Health, Bethesda, MD). To maintain consistency, each image was processed in the same manner including maintaining consistent light intensities during optical density measurements. The operations (Contrast-Limited Adaptive Histogram Equalization commands) included background subtraction (blocksize 100), mean filter (bin 256) and local contrast (maximum slope 2.50).

### Statistical analysis

Statistical analyses were carried out using commercial software (Prism, Irvine, CA; Excel, Microsoft, Redmond, WA). Data were expressed as means ± standard error of the mean (SEM) of samples characterized in three 2-week sample sets plated in duplicate or quadruplicate, and assayed in duplicate (n = 12 or n = 24, respectively). A two-way analysis of co-variance (ANCOVA) with a Bonferonni post-test (direct comparison with controls), Dunnett’s test (pairwise comparisons) and non-linear regression (polynomial quadratic function) were applied to statistically compare the treatment groups over time, with asterisks of *P < 0.05, **P < 0.01, and ***P < 0.001 indicating varying levels of statistical significance as noted in the figures.

## Results

**Figure 1.**
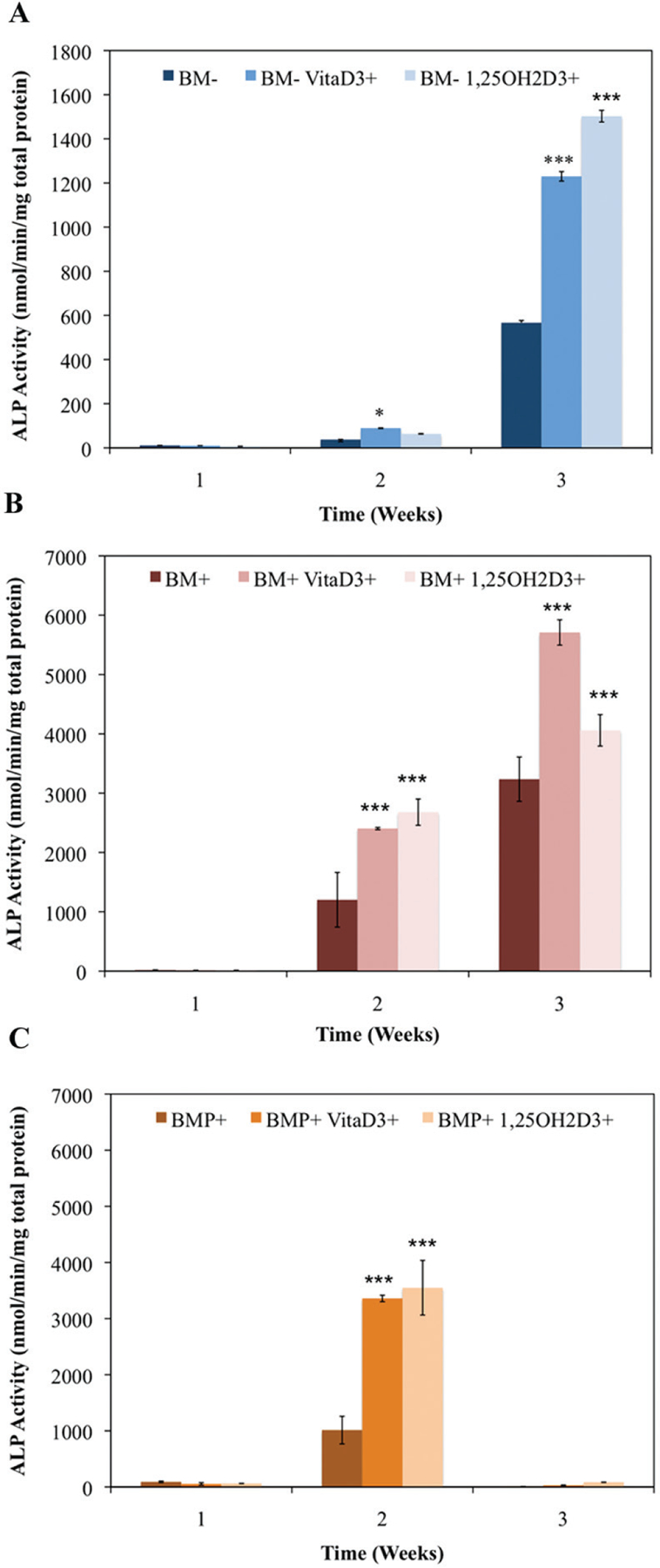
Temporal alkaline phosphatase activity of OPC1 treated with (A) ethanol vehicle control in standard bone medium (BM−), BM− containing vitamin D_3_ (BM− vitaD3+) and BM− with 1,25OH_2_D_3_ (BM− 1,25OH2D3+); (B) ethanol control in osteogenic medium (BM+) containing ascorbic acid, β-glycerophosphate and dexamethasone, BM+ containing vitamin D_3_ (BM+ vitaD3+), and BM+ with 1,25OH_2_D_3_ (BM+ vitaD3+); (C) ethanol control in BM+ containing rhBMP (BMP+), BMP+ containing vitamin D_3_ (BMP+ vitaD3+), and BMP+ with 1,25OH_2_D_3_ (BMP+ vitaD3+). Cellular isolates were collected on day 7, 14 and 21 during the differentiation time course. Treatment with both vitamin D metabolites significantly influenced OPC1 ALP activity in all medium groups (P < 0.01).

Increased ALP activity is known to be associated with osteoblast function and the matrix maturation stage of bone development. ALP activity here was found to be statistically higher (P < 0.01) in groups cultured in standard bone medium (BM−) containing vitaD_3_ by week two of culture, and significantly higher (P < 0.001) in groups containing either vitaD_3_ or 1,25OH_2_D_3_ by week three compared to the BM-ethanol vehicle control (Fig. 1A). For groups in osteogenic medium (BM+) both vitamin D metabolites displayed statistically higher (P < 0.001) ALP activity compared to the BM+ EtOH control in both the second and third week (Fig. 1B). All groups cultured in BM+ were significantly higher (P < 0.001) than the groups cultured in BM− during the 3-week culture period, and reached maximal ALP, while groups in BM− did not reach maximal ALP activity. Groups cultured in BM+ containing rhBMP (BMP+) showed a significant increase (P < 0.001) only in the second week compared to the BMP+ EtOH control, and by week three all groups displayed low levels of ALP activity (Fig. 1C). While groups cultured in BM+ continued to increase in ALP activity throughout the 3-week culture period, BM+ reached a peak in between weeks 1 and 3, and began to decline in between weeks 2 and 3.

**Figure 2.**
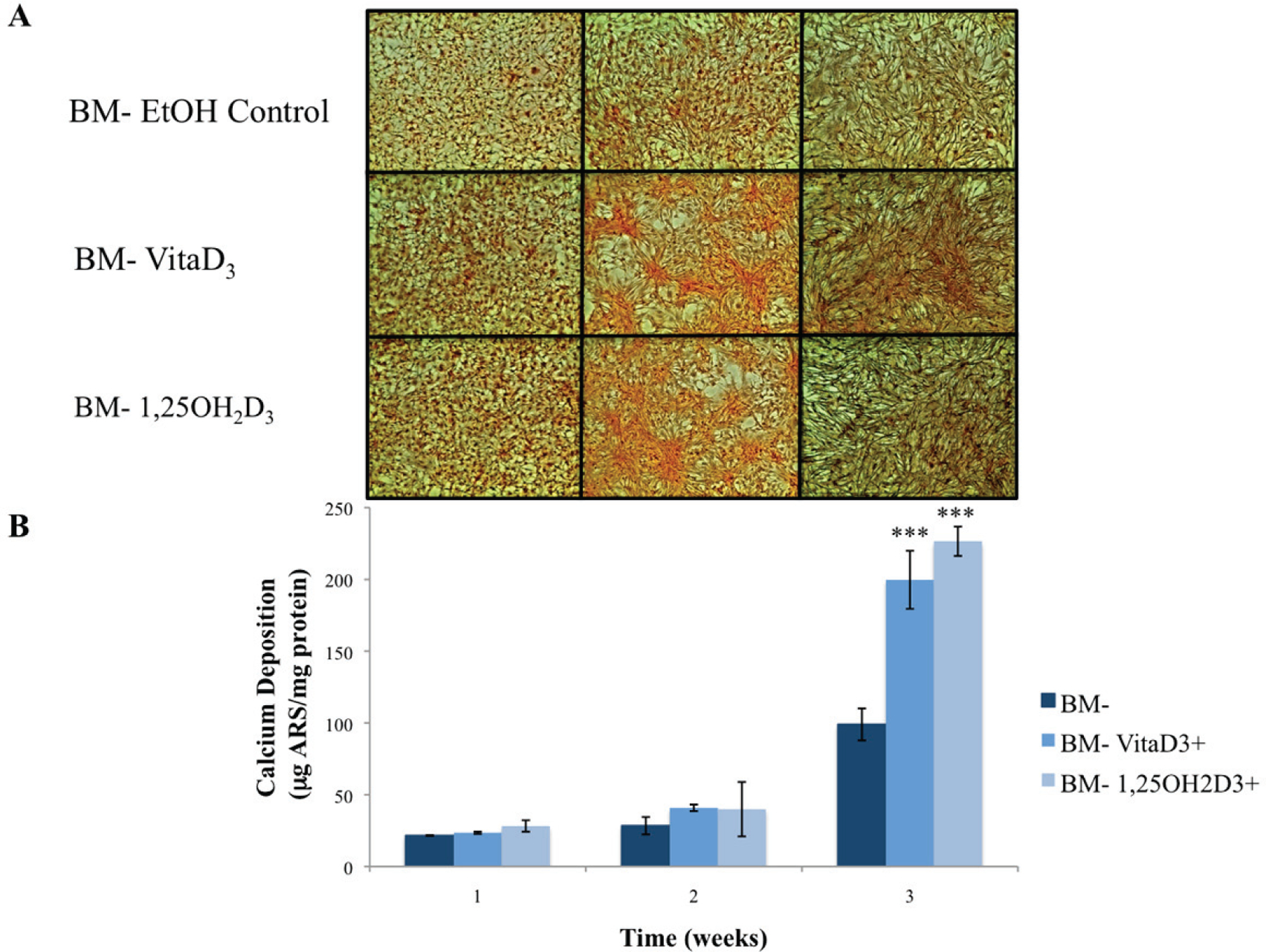
(A) Calcium deposition stained with alizarin red stain (ARS) of OPC1 cultured in BM− and either vitamin D metabolites, vitamin D_3_ or 1,25OH_2_D_3_. (B) After microscopic analysis, ARS was extracted from each culture well, and quantified on a microplate reader. By week three of culture, there was a statistically larger amount of calcium deposition detected by ARS in cells treated with either vitamin D_3_ or 1,25OH_2_D_3_ compared to that of the ethanol control (***P < 0.001).

**Figure 3.**
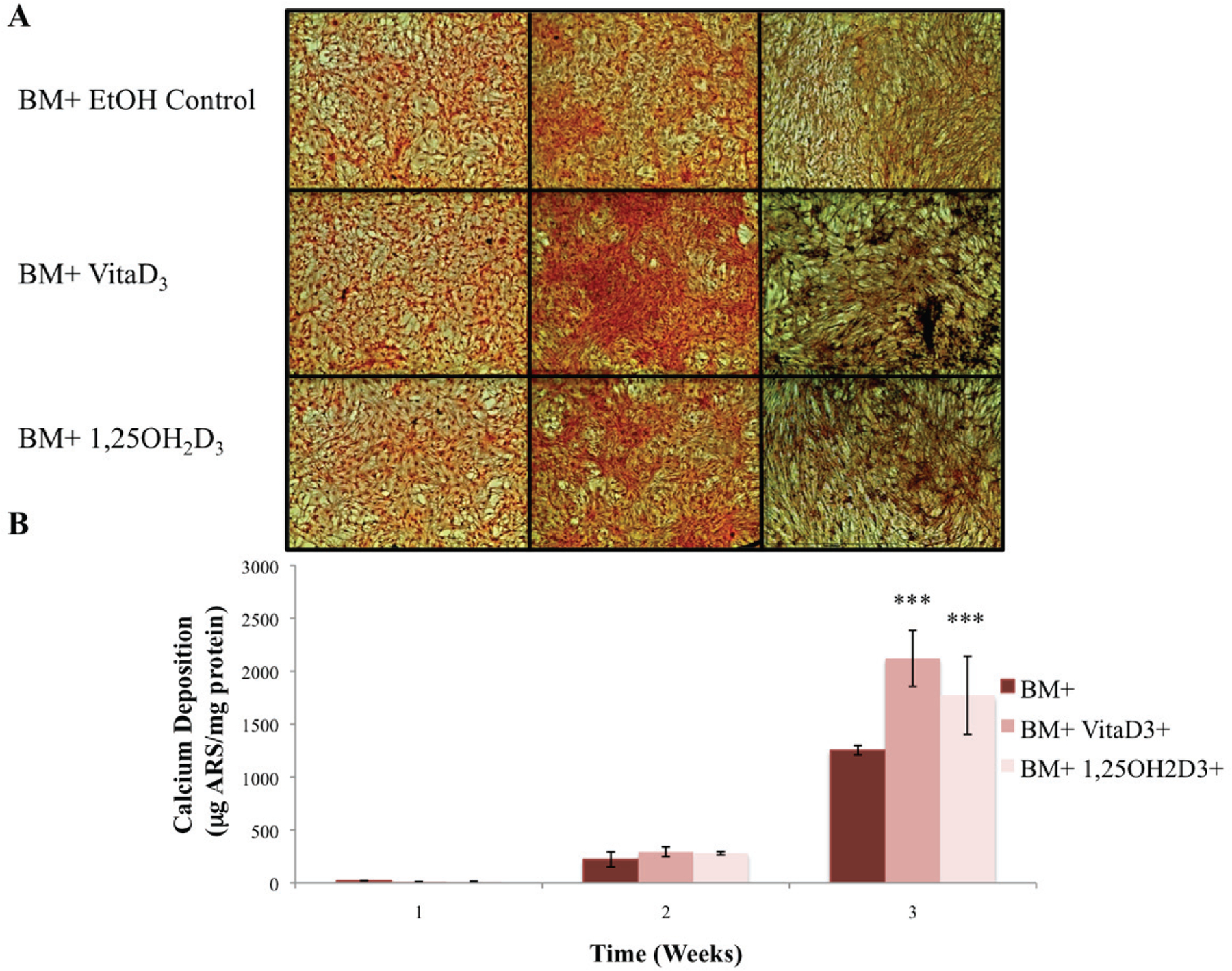
(A) OPC1 calcium deposition stained with ARS during culture in BM+ and either vitamin D_3_ or 1,25OH_2_D_3_. (B) After microscopic analysis, ARS was extracted from each culture well and read on a microplate reader. By week three of culture, there again was a larger amount of calcium deposition detected by ARS in cells treated with either vitamin D_3_ or 1,25OH_2_D_3_ compared to that of the ethanol control (***P < 0.001). In addition, there was more calcium deposition in the BM+ treatment groups compared to all BM− groups (shown in Fig. 2).

**Figure 4.**
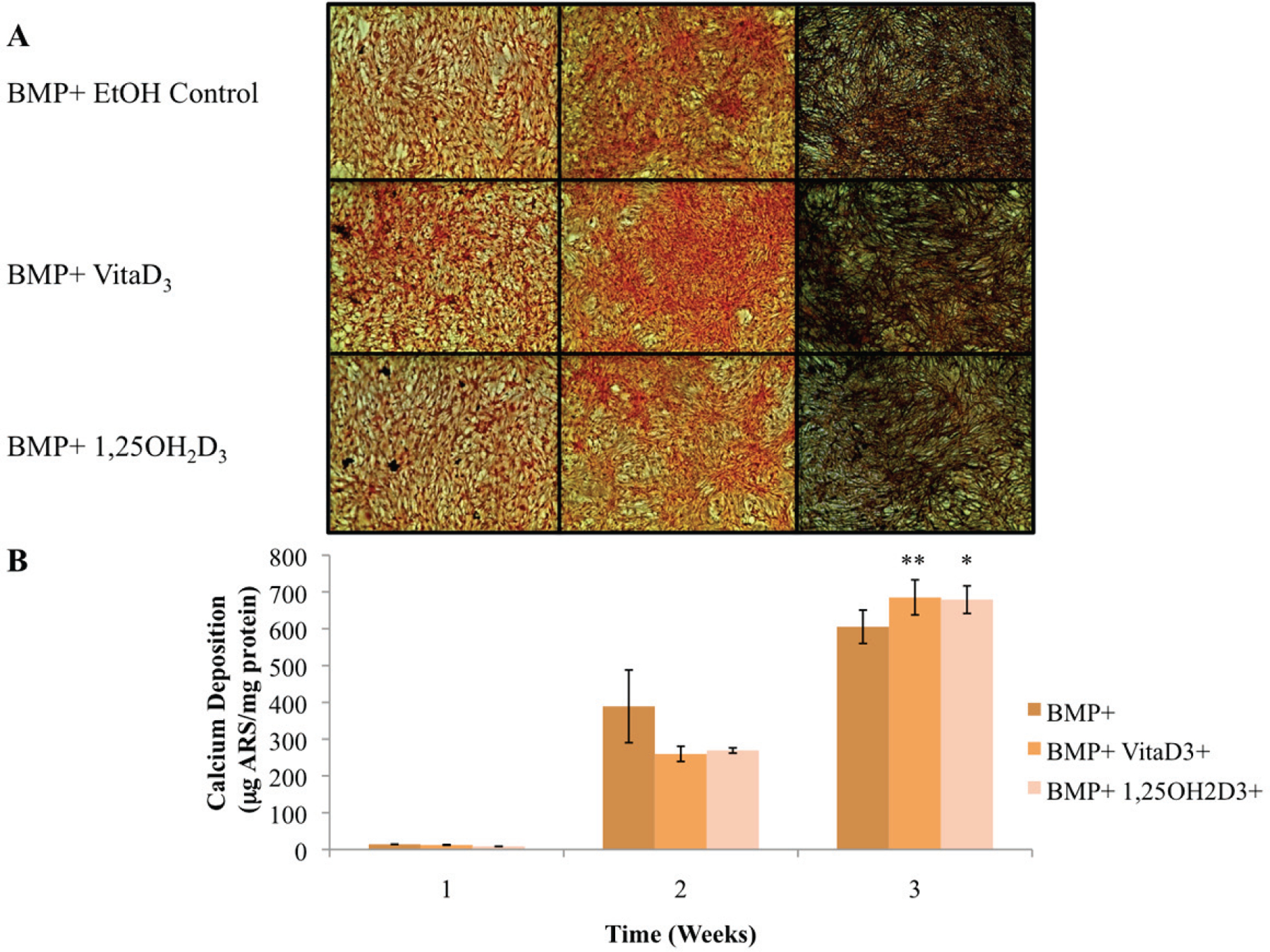
(A) OPC1 calcium deposition as stained with ARS during culture in BMP+ containing rhBMP-2 (BMP+) and either vitamin D_3_ or 1,25OH_2_D_3_. (B) Quantified ARS as extracted from each culture well and read on a microplate reader. In the second week of culture, the BMP+ EtOH control had a larger amount of calcium deposition in comparison with the treatment groups (P < 0.05). By week three of culture, there was more calcium deposition detected by ARS in cells treated with either vitamin D metabolites compared to that of the ethanol control (*P < 0.01). In addition, there was a statistically more calcium deposition in the BMP+ treatment groups (P < 0.001) compared to all BM− groups (plotted in Fig. 2).

The primary function of osteoblasts is to produce and maintain mineralized ECM. For our samples cultured in BM− with or without vitamin D metabolites, vitaD_3_ and 1,25OH_2_D_3_, calcium deposition was significantly greater (P < 0.001) in cultures containing either of the vitamin D metabolites compared with the BM− ethanol control (Fig. 2). OPC1 cultured in BM+ with or without the vitamin D metabolites showed a significantly higher amount of calcium deposition (P < 0.001) throughout the 3-week culture period compared to that of cultures in BM−. Cultures containing either vitaD_3_ or 1,25OH_2_D_3_ were significantly higher (P < 0.001) than the BM+ EtOH control by the third week of culture (Fig. 3). Cultures containing rhBMP (BMP+) showed a statistically significant amount of calcium deposition (P < 0.001) compared to that of the groups cultured in BM− throughout the 3-week culture period. The presence of vitaD_3_ in BMP+ medium caused a larger amount of calcium deposition (P < 0.01) compared to that of the BMP+ EtOH control, and slightly more deposition (P < 0.05) when in the presence of 1,25OH_2_D_3_ (Fig. 4).

**Figure 5.**
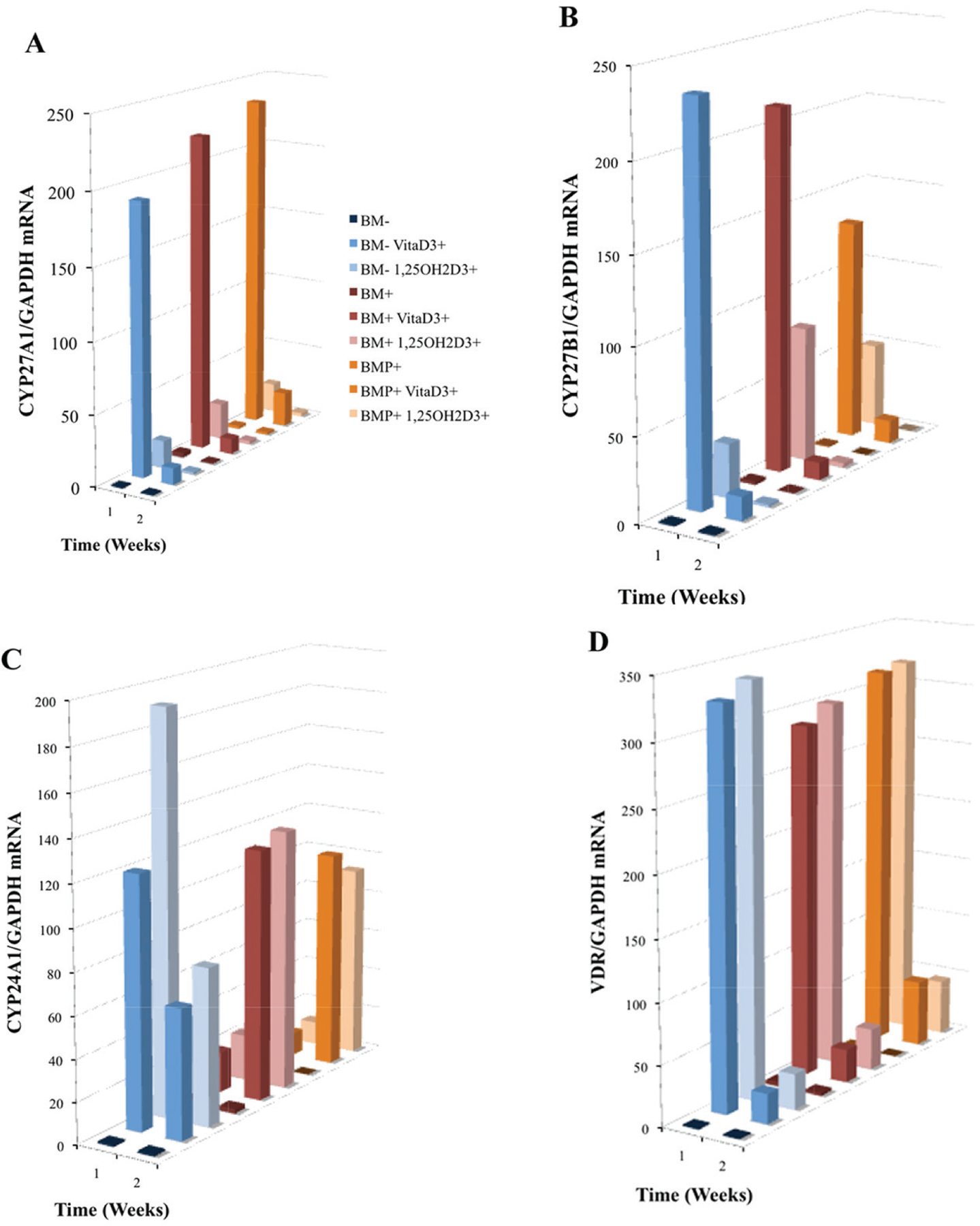
Induced mRNA expression of (A) CYP27A1, (B) CYP27B1, (C) CYP24A1 and (D) VDR in nine treatment group cultures of OPC1s including either vitamin D_3_ or 1,25OH_2_D_3_ in standard bone medium (BM−) and medium containing osteogenic factors (BM+ and BMP+). Total RNA was isolated and examined by quantitative RT-PCR. Data were normalized by the level of glyceraldehyde 3-phosphate dehydrogenase (GAPDH), a protein-coding gene. The figure legend for (A) applies to Fig. 5 and 6 where the bars (front to back) are associated with the listed experimental groups (top to bottom).

As mentioned, CYP27A1 and CYP27B1 are hydroxylases demonstrated to be involved in the metabolism of vitaD_3_ to 25OHD_3_, and convert 25OHD_3_ to 1,25OH_2_D_3_, respectively. Here, OPC1 expressed both CYP27A1 (Fig. 5A) and CYP27B1 (Fig. 5B) in response to the presence of vitaD_3_, relative to the BM− EtOH control. Increases in both hydroxylases occurred in some of the treatment within the first 72 h of treatment (treatment added on day 4 of culture) as measured at week one, but decreased dramatically by the second week in the continuous presence of vitaD_3_. In the groups containing osteogenic factors (BM+ and BMP+), the expression of CYP27A1 was higher then in the BM− group treated with vitaD_3_. However, in groups treated with BMP+, CYP27B1 expression was lower then in the BM− and BM+ groups treated with vitaD_3_. OPC1 also expressed CYP24A1 and VDR mRNA in response to vitaD_3_ and 1,25OH_2_D_3_ (Fig. 5C, D). In the first week, all groups treated with the vitamin D metabolites had significant increases (P < 0.001) in both CYP24A1 and VDR expression relative to the BM− control. BM− cultures treated with vitaD_3_ expressed a much higher amount of CYP24A1 mRNA relative to BM− control, compared to the cultures containing osteogenic factors. After the continuous presence of vitaD_3_, in the second week of culture, CYP24A1 expression decreased for the BM− groups, but increased in groups containing BM+ and BMP+. All groups treated with vitaD_3_ and 1,25OH_2_D_3_ had statistically more VDR mRNA expression (P < 0.001) relative to the BM− control during the first week of culture, but which decreased by the second week.

**Figure 6.**
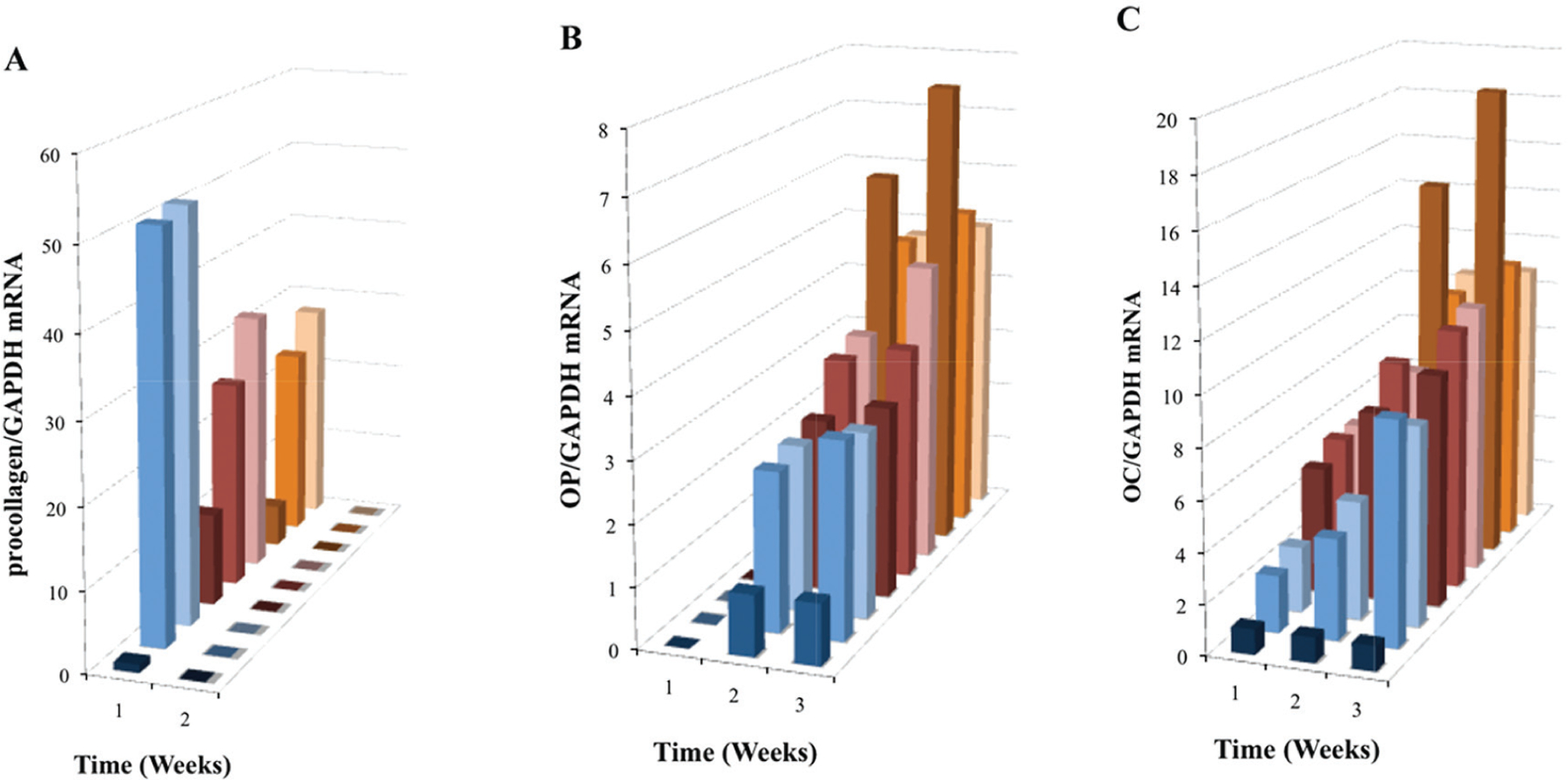
Induced mRNA expression of (A) pro*Col*I, (B) OP and (C) OC in nine treatment group OPC1 cultures containing the vitamin D metabolites and/or osteogenic factors (see the figure legend for Fig. 5A). Total RNA was isolated and examined by quantitative RT-PCR and data were again normalized by levels of GAPDH.

Synthesis of procollagen type I (pro*Col*I) is known to precede the production of collagen type I (*Col*I), a major component of bone ECM. This synthesis is enhanced during early osteoblast differentiation, while OC and OP are associated with later stages of osteoblast differentiation and bone development. Real-time PCR analysis showed a significant increase in pro*Col*I mRNA expression (P < 0.001) during the first week of OPC1 culture in all groups relative to the BM− control, and a higher increase with treatment groups containing either vitaD_3_ or 1,25OH_2_D_3_ (Fig. 6A). There was no statistically significant expression of pro*Col*I mRNA in the second week in any of the culture groups (P > 0.05). In contrast to the expression of pro*Col*I, OP mRNA expression was not seen in the first week of culture time in any of the groups and increased in both the second and third week of culture relative to the BM− control (Fig. 6B). The BMP+ control without vitamin D displayed the highest OP mRNA expression relative to the BM− control. Similar to OP mRNA expression, expression of OC mRNA was highest in the BMP+ control group, but was increased in all groups relative to the BM− control (Fig. 6C). However, in contrast to OP mRNA expression, OC mRNA expression was increased in the first week, and continued to increase during the 3-week culture period.

## Discussion

In this study, an engineered OPC1 was used to examine the effect of vitamin D metabolites, vitaD_3_ and 1,25OH_2_D_3_, on the maturation and mineralization of culture-expanded cells as a model for bone development. In comparison to the untreated cells, vitamin D metabolites in combination with ascorbic acid, β-glycerophosphate, dex and/or BMP-2, produced more calcium deposition and showed a greater increase in ALP over time. Cultures containing osteogenic factors varied in ALP activity depending on the presence of rhBMP-2. In addition to ALP activity and calcium deposition, mRNA expression of pro*Col*I, OC and OP were analyzed over the 3-week culture period, as well as the mRNA expression of genes associated with vitamin D action and metabolism.

Osteoblasts are bone-forming cells derived from multipotential progenitor cells within the bone marrow stroma that also are capable of committing to other mesenchymal lineages, including fibroblasts, myoblasts, chondrocytes, adipocytes and osteoblasts [3, 30]. Once precursors have committed to a specific lineage, tissue specific molecules are expressed. Phenotypical characteristics of an osteoblast include the synthesis and mRNA expression of proCol*I* preceding Col*I*, OC, OP, VDR, increased ALP activity and the ability to mineralize ECM. When osteoblast precursors commit to the osteoblastic lineage, the osteoblasts express bone matrix protein genes at different expression levels depending on the maturation level of the cells [31]. For example, immature mesenchymal cells and preosteoblasts weakly express *Col*I and collagen type II (*Col*II), while immature osteoblasts express OP, and bone sialoprotein (BSP), a component of mineralized tissues. Mature osteoblasts meanwhile strongly express OC [31] and may eventually become osteocytes when they are embedded into the bone matrix and express dentin matrix protein 1 (DMP-1).

*In vitro* human osteoblasts respond to exogenous 1,25OH_2_D_3_ by decreasing their rate of proliferation while increasing their expression of mRNA of osteogenic biomarkers such as OC and OP. While it has been known that circulating vitamin D is derived from the kidneys, recently a number of studies have demonstrated the extra-renal synthesis of 1,25OH_2_D_3_ from 25OHD_3_ in skin, liver, lymph nodes, activated monocytes/macrophages, dendritic cells and osteoblasts [11]. In contrast to extra-renal metabolism of 25OHD_3_ to 1,25OH_2_D_3_, there have been occasional reports of extra-hepatic metabolism of vitaD_3_, but no detailed analysis until now. Recently, we demonstrated that OPC1 could metabolize vitaD_3_ to 1,25OH_2_D_3_ in a dose-dependent manner [6], and in this present experiment, we were able to demonstrate the upregulation of mRNA expression of vitamin D associated hydroxylases CYP27A1, CYP27B1 and CYP24A1, as well as VDR in response to vitaD_3_. It was also demonstrated that vitaD_3_ influenced the expression of osteogenic mRNA pro*Col*I, OC and OP in the same manner as 1,25OH_2_D_3_.

In addition to the effects of vitamin D metabolites on OPC1, vitamin D in combination with common osteogenic factors was investigated. It was demonstrated that in combination with these osteogenic factors, matrix maturation and mineralization were significantly increased. However, there were differential results in bone maturation associated with ALP activity seen in cultures treated with BMP+. ALP expression is associated with bone cell phenotype, and in heavily mineralized cultures, cellular levels of ALP decline [32]. OPC1 cultured in BMP+ with or without vitamin D metabolites demonstrated an increase in mineralization, and consequently with the mRNA expression of OC and OP, proteins that are known to increase with the accumulation of mineralization, compared to the BM− and BM+ groups. These results indicate that rhBMP-2 had a major influence on OPC1 bone mineralization, that may exceed the influence of vitamin D. A major limitation to this result, was that BMP+ cultures were so heavily mineralized that by day 17 of culture time, the monolayer began to detach from the culture vessel.

It has been previously demonstrated that using dex, BMP-2 and 1,25OH_2_D_3_ tend to enhance a more differentiated osteoblast phenotype [33]. However in our study, OP and OC mRNA expression in groups treated with vitaD_3_ or 1,25OH_2_D_3_ and BMP+ was slightly lower than the BMP+ control, but higher than the osteogenic medium containing vitamin D metabolites and no BMP (BM+). This may be because we used a higher dose of 1,25OH2D3, which would also explain the very significant increase in catabolic enzyme mRNA expression of CYP24A1 seen in the second week of culture. In addition, it could be postulated that as a regulator of ECM mineralization, vitamin D dampened the effects of BMP. Many *in vitro* vitamin D studies investigating the antiproliferative and pro-differentiative effects of vitamin D are short-term and use a supra-physiological dose; however, for long-term cultures using vitamin D as an osteognenic factor, it may be beneficial to use a dose that more closely resembles the phsyiological dose.

It has been long recognized that long-term or excessive use of glucocorticoids (GC), whether from endogenous hypersecretion or iatrogenically, manifests adverse skeletal effects such as osteoporosis [34]. In human subjects receiving long-term GC treatment, bone loss is associated with the decrease in overall bone formation rate and in the mean thickness of the walls on newly synthesized trabecular bone, a pattern consistent with the decrease in number and/or activity of osteoblasts rather than the increase in number and/or activity of osteoblasts [34]. Paradoxically, several studies have demonstrated that treating cells derived from human bone with GCs promotes osteogenic differentiation, and has been proposed as a standard supplement for the *ex vivo* expansion of cells with osteogenic potential for use in tissue reconstruction and repair [34]. Dexamethasone is a GC that is commonly used as an inducer of bone marrow stromal cell differentiation, which reliably stimulates the development of many, but not all, phenotypic features of human osteoblasts [33]. However, GCs have deleterious effects *in vivo*, resulting in inhibition of osteoblast function. BMP-2 is another inducer of osteoblast differentiation, and is essential for postnatal bone formation. BMP-2 has been used to induce osteoblast differentiation *in vitro* in many human and animal models, and is believed to provide a more physiologic stimulus than dexamethasone; however, it appears to enhance a more mature osteoblast characteristic [33].

Overall, this work provided baseline two-dimensional culture data characterizing the interaction between vitamin D metabolites and an osteoprecursor cell line. Ongoing efforts are expanding this approach into a tissue engineered model created with three-dimensional culture and custom biomaterial scaffolds including the application of mechanical stimulation through access to a novel bioreactor [2]. In addition, the combined influence of vitamin D and biomechanics on isolated single cells within an optofluidic environment is concurrently being investigated. This active research theme addresses multiscale mechanotransduction through novel bioengineering approaches.

## Figures and Tables

**Table 1.** Oligionucleotide Single-Stranded Molecular Primers Used in Quantitative, Real-Time RT-PCR

Primer set	Forward	Reverse
CYP24A1	5’ - GCA GCC TAG TGC AGA TTT - 3’	5’ - ATT CAC CCA GAA CTG TTG - 3’
CYP27A1	5’ - GGC AAG TAC CCA GTA CGG - 3’	5’ - AGC AAA TAG CTT CCA AGG - 3’
CYP27B1	5’ - TGT TTG CAT TTG CTC AGA - 3’	5’ - CCG GGA GAG CTC ATA CAG - 3’
VDR	5’ - CCA GTT CGT GTG AAT GAT GG - 3’	5’ - GTC GTC GGT GAA GGA - 3’
OC/BGLAP	5’ - CTG GCC ACT GCA TTC TGC - 3’	5’ - AAC GGT GGT GCC ATA GAT GCG - 3’
OP/SPP1	5’ - AAA TAC CCA GAT GTG GC - 3’	5’ - AAC CAC ACT ACC TCG GC - 3’
Pro-*Col*I	5’ - TGA CGA GAC CAA GAA CTG - 3’	5’ - CCA AAG TCA CCA AAC CTA CC - 3’
